# Involvement of a Non-Human Sialic Acid in Human Cancer

**DOI:** 10.3389/fonc.2014.00033

**Published:** 2014-02-19

**Authors:** Annie N. Samraj, Heinz Läubli, Nissi Varki, Ajit Varki

**Affiliations:** ^1^Departments of Medicine, Pathology and Cellular and Molecular Medicine, Glycobiology Research and Training Center, University of California San Diego, La Jolla, CA, USA

**Keywords:** Neu5Gc, sialic acid, antibodies, inflammation, tumor antigen, red meat

## Abstract

Sialic acids are common monosaccharides that are widely expressed as outer terminal units on all vertebrate cell surfaces, and play fundamental roles in cell–cell and cell–microenvironment interactions. The predominant sialic acids on most mammalian cells are *N*-glycolylneuraminic acid (Neu5Gc) and *N*-acetylneuraminic acid (Neu5Ac). Neu5Gc is notable for its deficiency in humans due to a species-specific and species-universal inactivating deletion in the *CMAH* gene encoding the hydroxylase that converts CMP-Neu5Ac to CMP-Neu5Gc. However, Neu5Gc is metabolically incorporated into human tissues from dietary sources (particularly red meat), and detected at even higher levels in some human cancers. Early life exposure to Neu5Gc-containing foods in the presence of certain commensal bacteria that incorporate dietary Neu5Gc into lipooligosaccharides can lead to generation of antibodies that are also cross-reactive against Neu5Gc-containing glycans in human tissues (“xeno-autoantigens”). Such anti-Neu5Gc “xeno-autoantibodies” are found in all humans, although ranging widely in levels among individuals, and displaying diverse and variable specificities for the underlying glycan. Experimental evidence in a human-like Neu5Gc-deficient *Cmah^−^*^/^*^−^*mouse model shows that inflammation due to “xenosialitis” caused by this antigen–antibody interaction can promote tumor progression, suggesting a likely mechanism for the well-known epidemiological link between red meat consumption and carcinoma risk. In this review, we discuss the history of this field, mechanisms of Neu5Gc incorporation into tissues, the origin and specificities of human anti-Neu5Gc antibodies, their use as possible cancer biomarkers, implications of xenosialitis in cancer initiation and progression, and current and future approaches toward immunotherapy that could take advantage of this unusual human-specific phenomenon.

## Historical Background

Nearly 100 years ago, Hanganutziu and Deicher independently described human heterophile antibodies that agglutinated animal erythrocytes, as occurring in patients with serum-sickness, who had received therapeutic animal antisera (1, 2). Similar antibodies were later reported in patients with various diseases, despite no prior exposure to animal serum (3). Characterization of the antigenic determinants of these Hanganutziu–Deicher (H–D) antibodies occurred when Higashi et al. and Merrick et al. demonstrated that at least some of the major epitopes recognized were gangliosides containing a sialic acid (Sia) called *N*-glycolylneuraminic acid (Neu5Gc) (4, 5). This discovery spurred further research and H–D antibodies were also described in sera from patients with multiple pathological states such as rheumatoid arthritis, infectious mononucleosis, leprosy, syphilis, leukemia, Kawasaki disease, and various cancers (6–17). Expression of Neu5Gc on gangliosides and glycoproteins of human meconium and various human tumors was also detected by immunohistochemistry or thin-layer chromatography using polyclonal chicken antibodies raised against Neu5Gc-containing ganglioside GM3, or indirectly, via inhibition of bovine erythrocyte agglutination by a human H–D antiserum (10, 18–32). Given the data available at the time, it was reasonable to assume that Neu5Gc was an “oncofetal antigen,” expressed in fetal tissues, suppressed in adult life, and later up regulated during carcinogenesis. However, the evidence for an immune response raised questions about this assumption. In retrospect, the early reports of H–D antigens in cultured human cancer cell lines was probably due to animal serum used in the medium, and those in human cancer samples were likely of dietary origin (see below).

## Diversity in the Sialic Acids

**Figure 1:**
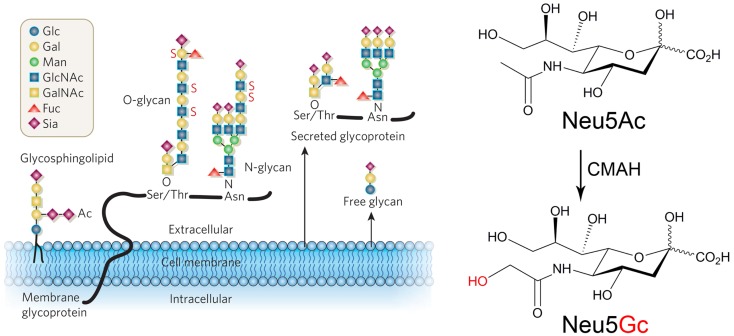
**Structures and predominant types of sialic acids**. Left panel: sialic acids (Sia) are often terminating units of *N*- and *O*-linked glycoproteins and glycosphingolipids that can be found on the cell surface as part of the glycocalyx, as well as on secreted glycoproteins. Ac, *O*-acetyl ester; Fuc, fucose; Gal, galactose; GalNAc, *N*-acetyl galactosamine; Glc, glucose; GlcNAc, *N*-acetylglucosamine; Man, mannose; Sia, sialic acid, type unspecified; S, sulfate ester. Right panel: the two main mammalian sialic acids Neu5Ac (*N*-acetylneuraminic acid) and Neu5Gc (*N*-glycolylneuraminic acid) differ by one oxygen atom, which is added by the enzyme cytidine monophosphate *N*-acetylneuraminic acid hydroxylase (CMAH) in the cytosol. Humans lack this enzymatic activity due to an inactivating mutation of the *CMAH* gene. Reproduced from Varki (34).

Classic studies of the structure, chemistry, and biosynthesis of sialic acids (Sias) occurred in parallel with the discovery and characterization of H–D antibodies. Sialic acids are a large family of nine-carbon-backbone monosaccharides primarily expressed in animals of the Deuterostome lineage (33, 34). They are most commonly found as the terminal units of the dense glycoconjugate coat covering cell surfaces, and of glycan chains attached to secreted molecules. By virtue of their position and high expression, Sias frequently act as the interface between the cell and the extracellular environment and mediate key roles such as stabilization of molecules and membranes via negative charge and hydrophilicity, transmembrane signaling, and receptor functions for self and non-self ligands (35, 36). They are also remarkably diverse owing to the various possible permutations involving different α-linkages between C-2 and the underlying glycan chain, as well as a variety of natural modifications. The predominant types of Sias in mammals include *N*-acetylneuraminic acid (Neu5Ac) and its hydroxylated version, Neu5Gc (Figure 1). These two molecules differ by a single oxygen atom, which is added to CMP-Neu5Ac in the cytosol, via a reaction catalyzed by the enzyme cytidine monophosphate *N*-acetylneuraminic acid hydroxylase (CMAH) (37–44). These activated sugars are then transported into the Golgi, where they act as donors for various sialyltransferases. (45).

## Human Deficiency of Neu5Gc Biosynthesis

Following the reports of Neu5Gc in fetal and malignant tissues, it was found that all humans are homozygous for an Alu-mediated deletion of exon 6 in the *CMAH* gene, which results in a truncated, inactive enzyme (46–48). We can only speculate if positive or negative selection was involved in the fixation of this mutation in the human lineage. Negative selection by a lethal infectious pathogen that preferred Neu5Gc as a binding target is a possibility (49). Positive selection due a fertility advantage to Neu5Gc-negative females could also have been operative (50). Alternatively, the human condition may simply be the result of a random mutation that became fixed in a small population that eventually gave rise to modern humans. Regardless of these considerations, this rather drastic change in the sialic acid topology of the cell surface can be dated back to ~2–3 mya, prior to the origin of the genus *Homo*, and may even have been involved in the origin of the genus (50). Not only has there been a corresponding increase in the precursor Neu5Ac, but also multiple related changes in Sia biology and pathogen regimes, which are discussed elsewhere (51, 52).

Since Neu5Gc has been found in many species of the deuterostome lineage (ranging from sea urchins to fish to non-human primates), the *CMAH* gene is at least 500 million years old (53). Notably, Neu5Gc deficiency seems to have evolved independently in sauropsids (birds and reptiles) and possibly in monotremes such as the platypus (53). Indeed, chickens are similar to humans in recognizing Neu5Gc as a foreign antigen and mounting a strong immune response against it. A serum-sickness like condition can be induced when horse serum is injected into chickens or by the virally induced Marek’s disease, a lymphoma that expresses Neu5Gc by unknown mechanisms (54–57).

## Neu5Gc Can be Metabolically Incorporated into Human Tissues from Dietary Sources

**Figure 2:**
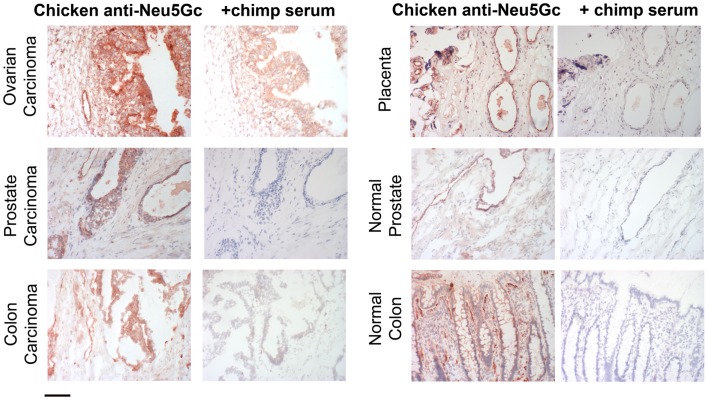
**Examples of incorporation of Neu5Gc in malignant and healthy human tissue**. Expression of Neu5Gc is observed to be enhanced in malignant epithelia as seen here in carcinomas of the ovary, prostate and colon (left panel). In contrast, expression of Neu5Gc in normal tissue is seen in the ducts of the prostate gland and in the epithelial lining of the colon (Right panel). Endothelial cells of the normal placenta is used here as a positive control for Neu5Gc immunostaining. As a negative control, the binding is blocked competitively with Neu5Gc-containing chimpanzee serum. Magnification used was 200× and scale bar is 100 μm.

Until the end of the last century, the evidence for Neu5Gc in human tissues remained indirect, based on polyclonal antibodies raised in chickens or using H–D antibodies from human patients (26, 32). In order to confirm the findings of the classic studies that showed the oncofetal expression pattern of Neu5Gc, a chicken anti-Neu5Gc IgY with high specificity and avidity was generated by affinity purification and it indeed detected accumulation of Neu5Gc in human tumors such as breast carcinomas, in fetal epithelial cells, and in placental endothelial cells (Figure 2) (58). Surprisingly, small but definite amounts of Neu5Gc were also detected in normal human secretory epithelia and on endothelia of small- and large-blood vessels. These findings were supported by mass-spectrometry analysis of purified sialic acids (58) and of *N*-glycans released from human tumor samples (59). Subsequent studies of *Cmah*^−/−^ mice showed a complete absence of Neu5Gc (59), indicating that there is no alternate pathway for the *de novo* biosynthesis of Neu5Gc. In the Absence of any other explanation, it was concluded that Neu5Gc must be entering human tissues exogenously via oral ingestion. Dietary sources that are rich in Neu5Gc include red meats such as beef, pork, lamb, and to a much lesser degree, cow’s milk products. Of significant note is the fact that plants and poultry do not contain Neu5Gc, and that fish samples studied so far contain low to trace amounts (58, 60).

Given that Neu5Gc incorporation is practically significant, there is a need for the sensitive and specific detection of Neu5Gc in human and *Cmah^−^*^/^*^−^*mouse tissues, as well as in biotherapeutic products (see below). There are several immunological and chemical methods to detect such trace amounts of Neu5Gc. Table 1 summarizes the different methods with description of advantages and disadvantages of each. Immunodetection with a polyclonal anti-Neu5Gc IgY was among the first methods employed for the detection of Neu5Gc in tissues. Polyclonal antibodies are able to broadly detect epitopes with a terminating Neu5Gcα2-R unit, whereas the various monoclonal antibodies are very specific for different Neu5Gc-containing gangliosides. While such antibodies (and newer versions that are more specific) are commonly applied to immunohistochemical techniques now, certain caveats apply. Gangliosides can be lost in the paraffin-embedding process, therefore, frozen section analysis is preferred. Strong binding to endothelial Neu5Gc maybe used as a positive control and negative control can include competitive inhibition with a Neu5Gc-containing reagent such as 10% chimpanzee serum, mild periodate oxidation of the sialic acid chain, or sialidase treatment of tissue sections. Each approach has pros and cons and must be properly controlled.

**Table 1:** **Overview of methods to detect small amounts of Neu5Gc**.

Method	Methodological features	Comments^a^
Polyclonal anti-Neu5Gc chicken IgY	Ganglioside GM3(Neu5Gc) as immunogen (54)	Recognizes gangliosides with Neu5Gcα2-R terminus. Applied to immunohistochemistry and TLC overlays
Affinity-purified polyclonal anti-Neu5Gc chicken IgY	Above polyclonal affinity purified on octyl-sepharose immobilized GM3(Neu5Gc) (157)	Specificity as above, likely cleaner background
Improved affinity-purified polyclonal anti-Neu5Gc chicken IgY	Above polyclonal antibody affinity purified on sequential columns of immobilized human and chimpanzee serum sialoglycoproteins, then eluted with Neu5Gc (158). Original immunogen noted to include bovine serum albumin as stabilizer – actually contaminated with Neu5Gc-rich bovine serum sialoglycoproteins, which acted as additional immunogens	Recognizes terminal Neu5Gc irrespective of linkage or underlying glycan chain (128). Applicable to ELISA, western blot, immunohistochemistry, and flow cytometry. Still contains IgY against animal albumin, removable by adsorption with column of mild periodate-borohydride-treated BSA (unpublished observations)
Monoclonal anti-Neu5Gc chicken IgYs	Monoclonal chicken anti-Neu5Gc IgYs (mChGc6-1, mChGc2-7) (74, 159)	mChGc2-7 has relatively broad specificity, similar to above polyclonal (128). Not yet studied for most applications
Monoclonal anti-Neu5Gc mouse/human IgM or IgG	MK2-34 murine IgM anti-GM2(Neu5Gc) (160), Y-2-HD-1, GMR14 murine IgG anti-GM2 (Neu5Gc) (81), 14F7 murine IgG anti-GM3(Neu5Gc) (140), GMR8 murine IgG anti-GM3(Neu5Gc) (161), GMR3 murine IgG anti-GD3(Neu5Gc-Neu5Gc-) (162), YK-3 human IgM against Neu5Gc disialogangliosides (163)	Each specific for different Neu5Gc gangliosides, but will miss many other Neu5Gc epitopes. Gangliosides get extracted from tissues during paraffin-embedding process and can be missed
Detection of sialidase or acid-released free Neu5Gc by HPAE-PAD	High-performance anion-exchange chromatography with pulsed amperometric detection (HPAE-PAD) (164–166)	Purification of released Neu5Gc improves background. Alkaline HPAEC conditions destroy *O*-acetyl groups
Detection of sialidase or acid-released free Neu5Gc by mass spectrometry	RP-HPLC with tandem MS interfaced with an electrospray ionization source (ESI) (167, 168)	Purification of released Neu5Gc and selective ion monitoring in mass spectrometry improves signal to noise
Detection of derivatized free Neu5Gc (sialidase or acid-released)	Fluorescent labeling with 1,2-diamino-4,5-methylenedioxybenzene (DMB) followed by RP-HPLC (164, 169, 170)	Sensitivity of fluorescence labeling can detect in the pmol range and ultra HPLC (UHPLC) allows run times as short as 10 min. DMB derivatization less likely to change Sia O-acetylation
Mass-spectrometry of glycans carrying Neu5Gc	Released *N*- or *O*-Glycan mixtures permethylated to facilitate purification and enhance signal in MALDI-TOF mass spectrometry (59)	Requires specific release of glycans. Alkaline permethylation destroys *O*-acetyl groups
Neu5Gc specific aptamers	Aptamers screened from a chemically synthesized nucleic acid library by “systematic evolution of ligands by exponential enrichment” (SELEX) (171)	Novel method with high affinity and apparent specificity Not tested on biological samples yet

*^a^See text for further discussion*.

## Mechanisms for Metabolic Incorporation of Neu5Gc into Cmah Null Cells and Tissues

*In vitro* evidence shows that cultured human epithelial cells can incorporate Neu5Gc into endogenous glycoproteins from animal products (such as fetal calf serum) in the medium. Incorporation of Neu5Gc involves fluid-phase pinocytosis to enter the lysosome, where a sialidase releases Neu5Gc from glycoconjugates, and a sialic acid transporter then delivers the foreign sialic acid to the cytosol. Free Neu5Gc in the extracellular fluid can follow the same pathway (61). Cytosolic Neu5Gc now becomes available for activation to CMP-Neu5Gc in the nucleus and is subsequently transported into the Golgi. Evidently, the single oxygen atom difference between the foreign Neu5Gc and the native Neu5Ac remains permissive for the human sialic biosynthetic machinery, which utilizes Neu5Gc as if it were a “self” molecule (61, 62).

With regard to the intact organism, few studies had investigated the fate of orally ingested Neu5Gc in mammals. Nöhle and Schauer first showed that radioactive free sialic acid fed to mice and rats appeared in urine and was metabolized, as a portion of the radioactivity was recovered as expired CO_2_ (63–65). Radioactivity from labeled sialic acids in mucins was also detected in murine tissues after oral gavage (65). Oral ingestion studies in normal adult humans showed that free Neu5Gc can be absorbed and excreted within 4–6 h and detected only in trace amounts in salivary mucins and facial hair (58). The generation of a *Cmah^−^*^/^*^−^*mouse in 2007 paved the way to further investigate the consequences of Neu5Gc deficiency (66). Using this human-like Neu5Gc-deficient mouse, it was shown that this foreign Sia can indeed masquerade as a self-molecule and be incorporated in normal healthy tissues, into the fetus, and into cancers (67). In these mice, free Neu5Gc (i.e., the monosaccharide) can be rapidly absorbed into circulation with peak levels seen at 1–2 h post ingestion and is efficiently excreted by the kidneys. In contrast, oral ingestion of glycosidically bound Neu5Gc (i.e., Neu5Gc-glycoconjugates such as porcine submaxillary mucins) led to incorporation into the small intestinal wall, liver, kidney, and was detectable in the peripheral circulation for several hours (67). Further investigation is necessary to understand the factors affecting the bioavailability of ingested Neu5Gc, the mechanisms behind the selective incorporation of glycosidically bound Neu5Gc, the preferential incorporation into endothelial and epithelial cells, and the eventual metabolic fate of this foreign molecule in human cells. With regard to the latter issue, recent work has revealed a degradative pathway involving sequential conversion to *N*-glycolylmannosamine (ManNGc), *N*-glycolylglucosamine (GlcNGc), and GlcNGc 6-phosphate, with eventual release of the glycolyl group into cellular metabolic pathways (68). With the exception of the last step, all other reactions are reversible, and little is known about rates of turnover and any other metabolic pathway that may be involved. The same studies also practically ruled out prior suggestions that Neu5Gc expression in humans might originate from an alternate pathway involving glycolyl-CoA (69, 70).

## Origins and Implications of the Human Immune Response Against Neu5Gc

Until the end of the 1990s, anti-Neu5Gc antibodies were thought to be present only in humans who had received therapeutic animal sera injections or those diagnosed with diseases like cancer, but not in normal healthy individuals. However, reports from the xenotransplantation field identified non-Gal antibodies specific for Neu5Gc that were detectable by flow cytometry in the majority of normal healthy humans (71). Parallel studies in our lab showed that the immune response against Neu5Gc was actually universal in humans (58), but of widely ranging levels. The antibodies were also diverse and of broad polyclonal specificities, arising from selective recognition of the underlying glycan as well (72, 73). The discrepancy with the earlier studies was likely due to the different methods of detection and/or antigens used in the assays. One of the cancer-associated H–D antigens used as the ELISA detection target in earlier studies was the ganglioside GM3(Neu5Gc) (4, 74, 75), an antigen against which antibodies in healthy humans tend to be low. Also, in some studies the antibody binding signal in normal subjects was simply assumed to be non-specific and subtracted as background (30, 76). Further, it was assumed that H–D antibodies were directed against Neu5Gc alone. However, a monosaccharide by itself cannot fill the binding pocket or paratope of the antibody, which can accommodate four to five sugars (77). Thus, the wide range of naturally occurring Neu5Gc-glycoconjugates would warrant a diverse, polyclonal immune response not just against the Sia, but also the underlying glycan (72). Indeed, chemo-enzymatically synthesized α2-3 or α2-6 linked sialoside pairs (i.e., identical glycans with either Neu5Gc or Neu5Ac) (78, 79) were used to analyze anti-Neu5Gc antibodies, revealing complex patterns of IgG, IgM, and IgA reactivities with marked variations in the antibody profiles within the normal human population (73). In retrospect, this also explains prior successes in obtaining monoclonal antibodies with specificities for particular Neu5Gc-containing molecules (74, 80–84).

The levels of these naturally occurring polyclonal antibodies can be quite high in some humans, approaching those of the major anti-glycan antibodies in normal human serum: anti-ABO blood group and anti-α-Gal (anti-Galα1-3Galβ1-4GlcNAc) (72). Such antibodies arise from antigenic stimulation by gastrointestinal bacteria and the latter are well known as an immunological barrier for xenotransplantation. In contrast to α-Gal, which is not expressed on human cells, the “xenogenic” Neu5Gc is enriched in cancer cells and can be incorporated into normal vertebrate cells as it is recognized by the biochemical pathways as a self or “autogenic” entity. Thus, Neu5Gc is the first example of a “xeno-autoantigen” (59, 62). Given this incorporation, circulating anti-Neu5Gc antibodies become potentially relevant in the pathogenesis of diseases associated with chronic inflammation such as cancer, atherosclerosis, and autoimmune disease (73, 85).

It was recently shown that anti-Neu5Gc antibodies in humans emerge during the first year of life and are likely not germ-line encoded “natural” antibodies (86). They appear at 6 months and steadily rise to adult levels by 1 year of age. This occurs coincident with the introduction of Neu5Gc via cow’s milk in baby formula. *Cmah^−^*^/^*^−^*mice were used to study this temporal effect further. A variety of Neu5Gc-feeding paradigms failed to generate anti-Neu5Gc antibodies in these mice. Instead, it was found that commensal non-typeable *Haemophilus influenza* is able to scavenge free dietary Neu5Gc and express it as an immunogenic epitope, thereby effectively “vaccinating” the host to generate anti-Neu5Gc IgM and IgG antibodies (86). It is possible that other commensal or pathogenic bacteria can also carry out this “xeno-autoimmunization,” and it is likely that there are other routes of immunization as well, considering the variable response that is seen across normal individuals. Further investigation is necessary to define the cellular and molecular pathways required for antibody selection, generation, and secretion, the specific B cell populations responsible, the levels of IgA antibodies in secretions, whether the anti-Neu5Gc response is T cell independent, and whether affinity maturation of the antibody binding site occurs following class-switching.

## Red Meat Consumption Increases Carcinoma Risk

Diet remains one of the most modifiable risk factors for many chronic diseases including cancer. Although meat is a valuable source of essential amino acids, iron, key vitamins and minerals, numerous epidemiological studies agree that consumption of red meat (meat from mammalian sources such as lamb, pork, and beef), is associated with not only increased risk of certain cancers but also cardiovascular disease (87, 88). Prominent among the large prospective cohorts are the Health Professionals Follow-up Study and the Nurses’ Health Study that recently confirmed that a higher intake of red meat was associated with a significantly elevated risk of cancer, cardiovascular disease, and total mortality (89).

The cancer type that has been most prominently associated with red meat consumption is colorectal cancer (CRC), one of the leading causes of cancer-related deaths in the United States (90). A statistically significant increase in CRC risk of 1.35-fold is seen with a high intake (>160 g/day) of red meat (91). This association is stronger for processed meat than for unprocessed meat. Esophageal, gastric, prostate, and endometrial cancer risk is also significantly elevated by high meat consumption, although conflicting reports exist (92–95). These associations are understandably variable, as diet is difficult to measure accurately, especially given the multiple correlations between different components of food.

There have been several mechanisms proposed by which red meat consumption increases the risk of cancer including: (1) high-fat intake (96); (2) the production of heterocyclic amines and polycyclic aromatic hydrocarbons formed by high temperatures during grilling (97); (3) the presence of mutagenic *N*-nitroso compounds (98); and, (4) the higher levels of heme iron as a promoter of carcinogenesis through increased cellular proliferation, increased oxidative stress, and iron-induced hypoxia signaling (99). The first three mechanisms have been effectively ruled out by epidemiological data, and by the fact that the risk is exclusive for red meat, and not for poultry or fish. More recently, a new mechanism for the link between cardiovascular disease and red meat was proposed to involve the intestinal microbiota. Metabolism of l-carnitine that is abundant in red meat by the microbiota generates trimethylamine-*N*-oxide (TMAO), which is proatherogenic in mice (100) and is associated with increased cardiovascular risk in humans (101). However, no connection of TMAO to cancer risk was reported.

Overall, there is no theory that has been conclusively proven. We propose that “xenosialitis” or the interaction between the non-human Neu5Gc and circulating anti-Neu5Gc results in chronic inflammation that promotes carcinogenesis and atherogenesis. In this regard, red meat consumption and the “western diet” have been associated with increased circulating markers of inflammation in human population studies (102). Thus, Neu5Gc may be the missing link between red meat consumption and risk of cancer and cardiovascular disease, an association that so far appears unique to humans. Evidence supporting our hypothesis is presented in the further sections.

## Role of Inflammation in Cancer

The mammalian immune system recognizes and eliminates cells with non-native DNA, including transformed cells (103–108). During cancer progression, however, tumor cells can escape elimination by the immune system by being selected for low immunogenicity or by being able to inhibit immune cell activation and induce an immunosuppressive microenvironment (104, 109–111). Some of these pathways can be targeted for cancer immunotherapies (108, 112–114).

In contrast to this immunosurveillance function, inflammation and associated activation of the immune system can also promote cancer progression (115–117). Chronic inflammation due to infectious and non-infectious agents such as auto-inflammatory diseases and diet-induced metabolic syndrome is an important etiology for the development of cancer (116–118). In this regard, epidemiological analyses have confirmed that interference with inflammation using non-steroidal anti-inflammatory drugs including aspirin are protective for the development of inflammation-induced cancers such as colorectal carcinomas (119, 120). Aspirin use has also been associated with reduced incidence of other cancers including those of the esophagus and stomach (121).

Inflammation not only works as promoter during carcinogenesis (inflammation-induced cancer), but growing tumors that escaped immunosurveillance also induce an inflammatory response that can support cancer progression (cancer-related inflammation) (115, 122). In particular, cells from the myeloid lineage such as neutrophils and monocytes/macrophages support cancer progression (110, 123–125). Thus, while the immune system exerts considerable immunosurveillance to eliminate tumor cells, inflammatory pathways can be co-opted by tumor cells to promote cancer progression. Moreover, inflammation can also act as an oncogenic promoter during tumorigenesis, inducing DNA damage via reactive oxygen species.

## Experimental Studies of Neu5Gc-Induced Xenosialitis in Cancer

Aberrant cell surface glycosylation is a well-known characteristic of cancer and can be altered by the loss or gain of certain structures, the presence of truncated structures, accumulation of precursors, and the synthesis of novel structures (126). Increased cell surface sialylation is one such example. Not only is there an increase in the total sialic acid content, but also significant changes in their modifications such as the accumulation of Neu5Gc. This molecule preferentially accumulates in malignant tissue due to multiple mechanisms, including increased macropinocytosis (61), rapid growth rates, and up-regulation of the sialin transporter in response to hypoxia (127). In keeping with this, subcutaneous syngeneic tumors showed high levels of Neu5Gc incorporation when *Cmah^−^*^/^*^−^*mice drank water containing Neu5Gc (67), conditions under which normal tissues were not easily loaded.

This accumulation occurs in the presence of circulating xeno-autoantibodies against Neu5Gc-glycans. Different experimental models were used to ask if inflammation mediated by the anti-Neu5Gc immune response (xenosialitis) could influence tumor growth by affecting cancer-related inflammation (59, 73). *Cmah^−^*^/^*^−^* mice subcutaneously injected with the low metastatic B16F1 murine melanoma cell line that expresses Neu5Gc on its surface developed antibodies against Neu5Gc and the tumors then grew larger over time (59). Subcutaneously injected murine MC38 tumor cells also formed larger tumors in *Cmah^−^*^/^*^−^*mice when polyclonal mono-specific murine anti-Neu5Gc antibodies were transferred passively via intraperitoneal injection (59). The increase in tumor growth was associated with enhanced infiltration of cells of the innate immune system, suggesting a role of these cells in promoting effects.

Blunting cancer-related inflammation in the subcutaneous MC38 model with a cyclooxygenase-2 inhibitor repressed the effect of anti-Neu5Gc antibodies (59). However, when higher doses of human anti-Neu5Gc antibodies purified from commercially available human intravenous immunoglobulins (IVIG) were transferred passively into mice by intraperitoneal injection, the growth of subcutaneous syngeneic MC38 tumors was significantly inhibited (73). This finding indicates that Neu5Gc-containing glycans could potentially serve as targets for immunotherapy. Indeed, when Neu5Gc production in MC38 cells was silenced via siRNA targeting the *Cmah* transcript, inhibition by transferred Neu5Gc antibodies was blunted (73). The experimental evidence discussed here indicates that low levels of anti-Neu5Gc antibodies can support cancer progression by enhancing tumor-related inflammation via induction of “xenosialitis,” and that higher doses might possibly be used to target tumors that have high levels of Neu5Gc in their glycocalyx. Further studies of this issue are underway, including models where tumors in mice accumulate Neu5Gc from dietary sources, in a manner similar to the human condition.

## Circulating Anti-Neu5Gc Antibodies as Tumor Markers

Earlier studies discussed above had reported an increased incidence of H–D antibodies in patients with cancer. Sera of patients with cancer were therefore analyzed for the presence of anti-Neu5Gc antibodies with specificity toward different Neu5Gc-glycan epitopes (73). A sialoglycan microarray of over 70 chemo-enzymatically synthesized sialoglycans including unique pairs of Neu5Gc- and Neu5Ac-containing glycans were used to analyze the different binding properties of antibodies from patients with breast cancer versus controls in order to identify a classifier of 20 Neu5Gc-containing glycans (73). Using these 20 Neu5Gc-containing glycan targets, analysis of binding properties of antibodies in sera were further tested and validated in more breast cancer patients. The four most significant glycans were also able to differentiate between controls and patients with various carcinomas including prostate, ovary, endometrium, colon, lung, and pancreas (73). These findings suggest that anti-Neu5Gc antibodies can function as tumor markers. Notably one of the most significant cancer-associated epitopes was very similar to the known sialyl-Tn tumor antigen, except that Neu5Gc replaced Neu5Ac (73). A caveat to the use of glycan microarrays is that interpretation of the results should take into consideration the differences in producing, presenting, coupling, and detecting the glycans (128). Anti-Neu5Gc antibodies can also be analyzed by using Neu5Gc-rich natural glycoproteins (such as wild type mouse serum) or by coupling glycans to polymers such as polyacrylamide and probing with antibodies on an ELISA plate (72, 86). This less expensive, simpler method could be used as a screening tool, perhaps to monitor disease progression and/or therapeutic response. However, dilution effects on antibody binding and competition between different antibody classes also complicate such studies, and caution must be taken to avoid reagents of animal origin that might introduce Neu5Gc contamination. Population studies are necessary to identify correlations between anti-Neu5Gc antibody levels and carcinoma risk and progression.

## Implications for Cancer Prevention and Treatment

Given the evidence for Neu5Gc in human tissues along with circulating anti-Neu5Gc antibodies, the observed xenosialitis in mouse models could potentially play an important role in the development of cancer (59). Since the source of this foreign sialic acid is the diet, avoidance of Neu5Gc-rich food could possibly reduce cancer risk. Indeed, as discussed above, Neu5Gc-rich red meat increases the risk of inflammation-induced cancers including CRC (89) and our proposed xenosialitis model could be replicating this increased risk. Furthermore, the data predict that the reduction of Neu5Gc consumption could also be important during therapy of already established malignancies in order to interfere with cancer progression. Indeed, high intake of a “western” dietary pattern, rich in red meat, was associated with a higher risk of recurrence and mortality among patients with stage III colon cancer treated with surgery and adjuvant chemotherapy (129).

It is well described that tumor cells are aberrantly sialylated and that content of sialic acid on the surface of tumor cells significantly increase as compared to cells in healthy tissues (130, 131). The up-regulation of sialylation might also explain why ingested Neu5Gc preferentially accumulates in cancer tissues (58, 67). Aberrant sialylation includes an increase of the tumor antigen sialyl-Tn (132–136), which is uncommon in normal tissues (132, 137, 138) or cryptic due to O-acetylation of the sialic acid (133) in healthy tissue. As discussed before, analysis of anti-Neu5Gc antibodies by a sialoglycan microarray showed significant up-regulation of antibodies targeting Neu5Gc-sialyl-Tn suggesting that such antibodies might function as specific tumor biomarkers (73). If this epitope is indeed a relatively cancer-specific antigen, it could be potentially useful as a target for imaging as well as a therapeutic tool for drug delivery. As *in vitro* assays have shown that human antibodies purified from IVIG preparations against Neu5Gc-Tn antigen can activate antibody-dependent cellular and complement-dependent cytotoxicity (ADCC and CDC) (73), the epitope could potentially be directly targeted by anti-Neu5Gc-Tn antibodies.

Neu5Gc-containing gangliosides including GM3 were originally described as an epitope for H–D antibodies (4, 5) and (Neu5Gc)GM3 was found to be a tumor-associated antigen particularly in skin and breast cancer (139, 140). Vaccination with (Neu5Gc)GM3 along with the outer membrane protein complex of *Neisseria meningitidis* in proteoliposomes lead to antibody production in advanced stage breast cancer patients in a phase I study (141). Immunization of mice with (Neu5Gc)GM3 led to the isolation of specific antibody 14F7 (140), which was recently humanized and named racotumumab (142, 143). This monoclonal antibody is able to bind several malignant tissues including skin cancers, neuroectodermal tumors, genitourinary cancer, non-small cell lung cancer, and tumors of the gastrointestinal tract (144–148). Trials testing the efficacy of racotumumab are currently recruiting (e.g., NCT01598454, NCT01460472). But there is no attempt to control for dietary Neu5Gc intake by patients.

Similar approaches might be taken toward other Neu5Gc-glycan epitopes that are enriched in tumors. For example, vaccination with proteins containing Neu5Gc as terminal sugars could potentially boost an immune response against established tumors. For instance, self-assembling MUC1 with Neu5Gc-Tn antigen could be used to immunize patients with carcinomas (149, 150).

Notably, Neu5Gc is present as a component of some cancer therapeutic agents. Immunotherapy with monoclonal antibodies against other tumor epitopes such as targeting HER2 with trastuzumab, EGF receptor 1 with cetuximab, or CD20 with rituximab are well integrated in today’s cancer therapies (151). Antibodies are glycosylated and biotechnological production of such glycoproteins can involve Neu5Gc-rich media and/or non-human cells expressing Neu5Gc (152). Thus, it was previously shown that incorporation of Neu5Gc in cetuximab enhanced the formation of immune complexes and promoted drug clearance (153). Moreover, *Cmah^−^*^/^*^−^*mice injected with cetuximab reacted with an anti-Neu5Gc immune response (153). Thus, avoidance of Neu5Gc during production of glycoproteins might improve half-life of therapeutics and also reduce the immunogenicity and therefore has important implications in cancer therapy.

## Conclusions and Future Directions

**Figure 3:**
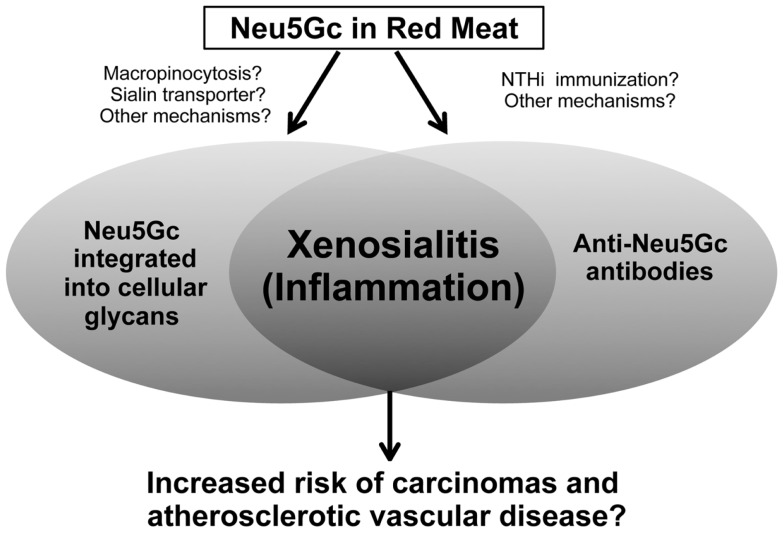
**Xenosialitis hypothesis**. Neu5Gc is metabolically incorporated from the diet (primarily red meats) into cellular glycans to form xeno-autoantigens. Anti-Neu5Gc antibodies or xeno-autoantibodies are induced by immunization via commensal bacteria (such as non-typeable *Haemophilus influenza* or NTHi) that scavenge and express Neu5Gc. The resulting antigen–antibody interaction is hypothesized to lead to chronic inflammation termed xenosialitis that potentially promote cancer and/or atherosclerotic vascular disease.

We have discussed the incorporation of the non-human sialic acid Neu5Gc into human tissues, and its potential impact on cancer initiation and progression. We particularly emphasized the resulting inflammatory activation or xenosialitis induced by xeno-autoantibodies against Neu5Gc-containing epitopes (Figure 3). Notably, Neu5Gc can also be incorporated into glycans of endothelial cells in healthy individuals and the subsequent xenosialitis has been potentially implicated in the pathogenesis and progression of atherosclerosis (85). Additionally, screening of antibody specificity by Neu5Ac/Neu5Gc specific sialoglycan microarray revealed a Neu5Gc antibody response in children with Kawasaki disease suggesting a possible role of these antibodies in disease progression (154). All these findings support the hypothesis that xenosialitis may be involved in various inflammatory diseases. Thus, avoidance of the Neu5Gc by reducing the consumption of Neu5Gc-containing food could not only have a major impact on the prevention of malignant diseases, but also potentially reduce the risk of cardiovascular disorders. Furthermore, examination and cloning of human anti-Neu5Gc xeno-autoantibodies can provide information about the nature of the anti-Neu5Gc immune response and offer ways to manipulate this in order to produce more efficient vaccines against Neu5Gc-containing tumor-associated epitopes.

Further analysis is needed to determine the pathways by which Neu5Gc is absorbed and transported to healthy and cancerous tissues, in order to be able to interfere with pathological consequences of Neu5Gc incorporation. Since Neu5Gc uses the same biochemical pathways as Neu5Ac, Neu5Gc could be potentially “flushed” out of the body by high amounts of Neu5Ac. The relatively specific accumulation of Neu5Gc on glycans of tumor cells, in particular as Neu5Gc-Tn antigen and (Neu5Gc)GM3 ganglioside is particularly intriguing and should be further studied to harness tumor cells for therapeutic and diagnostic purposes. However, it must be carefully determined if anti-Neu5Gc antibodies are well suited for immunotherapy of tumors or if there are “off-target” effects due to the presence of Neu5Gc-containing epitopes in other tissues. The accumulation of Neu5Gc-containing glycans in tumors in *Cmah^−^*^/^*^−^*mice fed with the monosaccharide Neu5Gc (67) suggests another strategy of deliberately loading tumors in a selective fashion in order to subsequently target them. Such “loading” of tumor tissue with Neu5Gc might be optimally combined with monoclonal antibody immunotherapies. Finally, it remains to be seen if the increase of Neu5Gc-containing sialoglycans on the surface of cancer cells might directly influence the cell intrinsic properties such as response to hypoxia or signaling through glycosylated surface receptors.

While much more work is needed to prove or disprove the overall hypothesis, it is encouraging that a prominent textbook of cancer biology has designated this as an “area to watch” (155). But major milestones of progress in the field are likely to be slow. While we are not assuming a similar level of importance to disease pathogenesis, it is instructive to compare the progress to date (Table 2) with those underlying the seminal discovery that cholesterol played a key role in atherosclerosis progression and risk (156). Cholesterol was found in atherosclerotic plaques in 1910, familial hypercholesterolemia was associated with early heart attacks in 1949, and population studies showed an association of heart disease with cholesterol levels by the early 1960s. However two more decades followed before cholesterol lowering by statins was shown to reduce heart attacks and another decade passed before a large scale double-blinded statin trial showed a positive effect in 1994 (156). While studies have clearly shown that Neu5Gc is a tumor-associated antigen with diagnostic and therapeutic potential and mouse models have established the role of xenosialitis in tumor progression, fundamental questions remain unanswered. Can anti-Neu5Gc antibodies and/or Neu5Gc tissue load predict carcinoma risk in human population studies? Will lowering Neu5Gc load reduce carcinoma risk? Is it possible to eliminate the foreign Neu5Gc from tissues or perhaps interfere with the metabolic incorporation process? Considering the potential impact on the prevention and treatment of human disease, it is evident that Neu5Gc and its interactions warrant further experimental and translational investigation.

**Table 2:** **Timeline of discoveries/studies concerning Neu5Gc and anti-Neu5Gc antibodies**.

Year(s)	Discoveries/studies	Reference
1924	Discovery of H–D antibodies in patients with serum-sickness	(1, 2)
1970–1980s	Association of H–D antibodies with multiple pathological states, including cancer	(3, 6–9)
1977–1978	Definition of Neu5Gc as key component of H–D antigen	(5)
1980s	Neu5Gc detected in human meconium and carcinomas by immunohistochemistry or TLC; assumed to be an “oncofetal” antigen	(10, 18–22)
1998	Human deficiency of Neu5Gc synthesis due to *CMAH* inactivation	(46–48)
2003	First results of trial with vaccine containing GM3 (Neu5Gc)	(141)
2003	Metabolic incorporation of Neu5Gc in human tissues from dietary sources	(58)
2003	Neu5Gc is enriched in red meats	(58)
2003	Anti-Neu5Gc response is universal to humans	(58, 71, 72)
2005	Molecular mechanisms of uptake and incorporation of Neu5Gc into cells elucidated – role of macropinocytosis and lysosomal sialin transporter noted	(61)
2006	Up-regulation of the sialin transporter in response to hypoxia in cancer increases Neu5gc accumulation	(127)
2007	Development of Cmah^−/−^mouse model. No alternate pathway for Neu5Gc synthesis	(66)
2008	Passively transferred anti-Neu5Gc antibodies enhance Cmah-positive carcinoma progression in Cmah^−/−^mice	(59)
2010	Origin of anti-Neu5Gc antibodies via “xeno-autoimmunization” by commensal bacteria that incorporate diet-derived Neu5Gc	(86)
2011	Humanized anti-Neu5Gc-GM3 antibody racotumumab, first trials using this antibody start recruiting	(143)
2011	Neu5Gc-sialyl Tn as potential biomarker cancer biomarker	(73)
2012	Oral glycosidically linked bound Neu5Gc preferentially incorporated into Cmah^−/−^mouse tissues, fetuses, and orthotopic tumors	(67)
2012	Intracellular degradative pathway for Neu5Gc discovered	(68)
2013	Simple method for assessment of human anti-Neu5Gc antibodies	(154)

*See text for discussion*.

## Conflict of Interest Statement

Ajit Varki and Nissi Varki are co-founders of and advisors to Sialix, Inc., which has licensed UCSD technologies related to anti-Neu5Gc antibodies in cancer. The other co-authors declare that the research was conducted in the absence of any commercial or financial relationships that could be construed as a potential conflict of interest.

## References

[1] Hanganutziu M Hémagglutinines hétérogénétiques après injection de sérum de cheval. CR Séances Soc Biol (1924) 91:1457–9

[2] Deicher H Über die Erzeugung heterospezifischer Hämagglutinine durch Injektion artfremden. Serums Z Hyg (1926) 106:561–79 10.1007/BF02176298

[3] Kasukawa R Kano K Bloom ML Milgrom F Heterophile antibodies in pathologic human sera resembling antibodies stimulated by foreign species sera. Clin Exp Immunol (1976) 25:122–32 825336 PMC1541393

[4] Higashi H Naiki M Matuo S Okouchi K Antigen of “serum sickness” type of heterophile antibodies in human sera: identification as gangliosides with N-glycolylneuraminic acid. Biochem Biophys Res Commun (1977) 79:388–95 10.1016/0006-291X(77)90169-3 412499

[5] Merrick JM Zadarlik K Milgrom F Characterization of the Hanganutziu-Deicher (serum-sickness) antigen as gangliosides containing N-glycolylneuraminic acid. Int Arch Allergy Appl Immunol (1978) 57:477–80 10.1159/000232140 78906

[6] Nishimaki T Kano K Milgrom F Studies on heterophile antibodies in rheumatoid arthritis. Arthritis Rheum (1978) 21:634–8 10.1002/art.1780210604 367377

[7] Nishimaki T Kano K Milgrom F Studies on immune complexes in rheumatoid arthritis. Arthritis Rheum (1978) 21:639–44 10.1002/art.1780210605 736995

[8] Nishimaki T Kano K Milgrom F Hanganutziu-Deicher antigen and antibody in pathologic sera and tissues. J Immunol (1979) 122:2314–8 109527

[9] Morito T Kano K Milgrom F Hanganutziu-Deicher antibodies in infectious mononucleosis and other diseases. J Immunol (1982) 129:2524–8 6183336

[10] Ikuta K Nishi Y Shimizu Y Higashi H Kitamoto N Kato S Hanganutziu-Deicher type-heterophile antigen-positive cells in human cancer tissues demonstrated by membrane immunofluorescence. Biken J (1982) 25:47–50 6982042

[11] Arita K Ikuta K Nishi Y Kato S Yamauchi E Maki S Heterophile Hanganutziu-Deicher antibodies in sera of patients with Kawasaki diseases. Biken J (1982) 25:157–62 6897861

[12] Takiguchi M Tamura T Goto M Kusakawa S Milgrom F Kano K Immunological studies on Kawasaki disease. I. Appearance of Hanganutziu-Deicher antibodies. Clin Exp Immunol (1984) 56:345–52 6375917 PMC1536237

[13] Mukuria JC Naiki M Hashimoto M Kato S A specific enzyme-linked immunosorbent assay (ELISA) procedure for detection of heterophile Hanganutziu and Deicher (HD) antibodies. J Immunol Methods (1986) 86:179–85 10.1016/0022-1759(86)90450-3 2418121

[14] Mukuria CJ Fujii Y Kato S Naiki M Specificities of human heterophile Hanganutziu and Deicher (HD) antibodies to glycosphingolipids and a glycoprotein. J Biochem (1986) 100:469–75 3491066 10.1093/oxfordjournals.jbchem.a121735

[15] Morito T Nishimaki T Masaki M Yoshida H Kasukawa R Nakarai H Studies on Hanganutziu-Deicher antigens-antibodies. I Hanganutziu-Deicher antibodies of IgG class in liver diseases. Int Arch Allergy Appl Immunol (1986) 81:204–8 10.1159/000234135 3095247

[16] Nakarai H Saida T Shibata Y Irie RF Kano K Expression of heterophile, Paul-Bunnell and Hanganutziu-Deicher antigens on human melanoma cell lines. Int Arch Allergy Appl Immunol (1987) 83:160–6 10.1159/000234349 3294601

[17] Higashihara T Takeshima T Anzai M Tomioka M Matsumoto K Nishida K Survey of Hanganutziu and Deicher antibodies in operated patients. Int Arch Allergy Appl Immunol (1991) 95:231–5 10.1159/000235434 1937925

[18] Ohashi Y Sasabe T Nishida T Nishi Y Higashi H Hanganutziu-Deicher heterophile antigen in human retinoblastoma cells. Am J Ophthalmol (1983) 96:321–5 6351622 10.1016/s0002-9394(14)77822-5

[19] Higashi H Fukui Y Ueda S Kato S Hirabayashi Y Matsumoto M Sensitive enzyme-immunostaining and densitometric determination on thin-layer chromatography of N-glycolylneuraminic acid-containing glycosphingolipids, Hanganutziu-Deicher antigens. J Biochem (1984) 95:1517–20 6378900 10.1093/oxfordjournals.jbchem.a134760

[20] Higashi H Nishi Y Fukui Y Ikuta K Ueda S Kato S Tumor-associated expression of glycosphingolipid Hanganutziu-Deicher antigen in human cancers. Gann (1984) 75:1025–9 6394416

[21] Mukuria JC Naiki M Hashimoto M Nishiura K Okabe M Kato S A potential radioimmunoassay system for detection of Hanganutziu-Deicher type heterophile antigen(s) and antibodies in tissues and fluids. J Immunol Methods (1985) 80:97–106 10.1016/0022-1759(85)90168-1 3874240

[22] Higashi H Hirabayashi Y Fukui Y Naiki M Matsumoto M Ueda S Characterization of N-glycolylneuraminic acid-containing gangliosides as tumor-associated Hanganutziu-Deicher antigen in human colon cancer. Cancer Res (1985) 45:3796–802 3874688

[23] Nowak JA Jain NK Stinson MW Merrick JM Interaction of bovine erythrocyte N-glycolylneuraminic acid-containing gangliosides and glycoproteins with a human Hanganutziu-Deicher serum. Mol Immunol (1986) 23:693–700 10.1016/0161-5890(86)90079-9 3099177

[24] Mukuria JC Naiki M Kato S Microstructure of the sialic acid moiety of N-glycolylneuraminyllactosylceramide and the elucidation of Hanganutziu and Deicher (HD) antigenicity. Immunol Lett (1986) 12:165–9 3487502 10.1016/0165-2478(86)90100-8

[25] Hirabayashi Y Higashi H Kato S Taniguchi M Matsumoto M Occurrence of tumor-associated ganglioside antigens with Hanganutziu-Deicher antigenic activity on human melanomas. Jpn J Cancer Res (1987) 78:614–20 3112076

[26] Hirabayashi Y Kasakura H Matsumoto M Higashi H Kato S Kasai N Specific expression of unusual GM2 ganglioside with Hanganutziu-Deicher antigen activity on human colon cancers. Jpn J Cancer Res (1987) 78:251–60 3106281

[27] Higashi H Sasabe T Fukui Y Maru M Kato S Detection of gangliosides as N-glycolylneuraminic acid-specific tumor-associated Hanganutziu-Deicher antigen in human retinoblastoma cells. Jpn J Cancer Res (1988) 79:952–6 10.1111/j.1349-7006.1988.tb00060.x 2460424 PMC5917607

[28] Kawachi S Saida T Uhara H Uemura K Taketomi T Kano K Heterophile Hanganutziu-Deicher antigen in ganglioside fractions of human melanoma tissues. Int Arch Allergy Appl Immunol (1988) 85:381–3 10.1159/000234536 3258290

[29] Fukui Y Maru M Ohkawara K Miyake T Osada Y Wang DQ Detection of glycoproteins as tumor-associated Hanganutziu-Deicher antigen in human gastric cancer cell line, NUGC4. Biochem Biophys Res Commun (1989) 160:1149–54 10.1016/S0006-291X(89)80123-8 2658991

[30] Nakarai H Chandler PJ Kano K Morton DL Irie RF Hanganutziu-Deicher antigen as a possible target for immunotherapy of melanoma. Int Arch Allergy Appl Immunol (1990) 91:323–8 10.1159/000235135 2354874

[31] Saida T Ikegawa S Takizawa Y Kawachi S Immunohistochemical detection of heterophile Hanganutziu-Deicher antigen in human malignant melanoma. Arch Dermatol Res (1990) 282:179–82 10.1007/BF00372619 2369143

[32] Kawachi S Saida T Analysis of the expression of Hanganutziu-Deicher (HD) antigen in human malignant melanoma. J Dermatol (1992) 19:827–30 1293171 10.1111/j.1346-8138.1992.tb03791.x

[33] Schauer R Sialic acids as regulators of molecular and cellular interactions. Curr Opin Struct Biol (2009) 19:507–14 10.1016/j.sbi.2009.06.003 19699080 PMC7127376

[34] Varki A Glycan-based interactions involving vertebrate sialic-acid-recognizing proteins. Nature (2007) 446:1023–9 10.1038/nature05816 17460663

[35] Varki A Schauer R Sialic acids. In: Varki A Cummings RD Esko JD Freeze HH Stanley P Bertozzi CR, editors. Essentials of Glycobiology. Cold Spring Harbor, NY: Cold Spring Harbor Laboratory Press (2009). p. 199–218

[36] Chen X Varki A Advances in the biology and chemistry of sialic acids. ACS Chem Biol (2010) 5:163–76 10.1021/cb900266r 20020717 PMC2825284

[37] Schauer R Schoop HJ Faillard H On biosynthesis of the glycolyl groups of N-glycolylneuraminic acid oxidative conversion of N-acetyl groups to glycolyl groups. Hoppe Seylers Z Physiol Chem (1968) 349:645–52 10.1515/bchm2.1968.349.1.645 5697796

[38] Buscher HP Casals SJ Schauer R Mestres VP Biosynthesis of N-glycolylneuraminic acid in porcine submandibular glands. Subcellular site of hydroxylation of N-acetylneuraminic acid in the course of glycoprotein biosynthesis. Eur J Biochem (1977) 77:297–310 10.1111/j.1432-1033.1977.tb11668.x 891536

[39] Shaw L Schauer R The biosynthesis of N-glycoloylneuraminic acid occurs by hydroxylation of the CMP-glycoside of N-acetylneuraminic acid. Biol Chem Hoppe Seyler (1988) 369:477–86 10.1515/bchm3.1988.369.1.477 3202954

[40] Muchmore EA Milewski M Varki A Diaz S Biosynthesis of N-glycoly neuraminic acid. The primary site of hydroxylation of N-acetylneuraminic acid is the cytosolic sugar nucleotide pool. J Biol Chem (1989) 264:20216–23 2684973

[41] Kozutsumi Y Kawano T Kawasaki H Suzuki K Yamakawa T Suzuki A Reconstitution of CMP-N-acetylneuraminic acid hydroxylation activity using a mouse liver cytosol fraction and soluble cytochrome b5 purified from horse erythrocytes. J Biochem (1991) 110:429–35 1769971 10.1093/oxfordjournals.jbchem.a123598

[42] Shaw L Schneckenburger P Carlsen J Christiansen K Schauer R Mouse liver cytidine-5’-monophosphate-N-acetylneuraminic acid hydroxylase – catalytic function and regulation. Eur J Biochem (1992) 206:269–77 10.1111/j.1432-1033.1992.tb16925.x 1587278

[43] Kawano T Kozutsumi Y Takematsu H Kawasaki T Suzuki A Regulation of biosynthesis of N-glycolylneuraminic acid-containing glycoconjugates: characterization of factors required for NADH-dependent cytidine 5’monophosphate-N-acetylneuraminic acid hydroxylation. Glycoconj J (1993) 10:109–15 10.1007/BF00731194 8358221

[44] Kawano T Koyama S Takematsu H Kozutsumi Y Kawasaki H Kawashima S Molecular cloning of cytidine monophospho-N-acetylneuraminic acid hydroxylase. Regulation of species- and tissue-specific expression of N-glycolylneuraminic acid. J Biol Chem (1995) 270:16458–63 10.1074/jbc.270.27.16458 7608218

[45] Shaw L Schauer R Detection of CMP-N-acetylneuraminic acid hydroxylase activity in fractionated mouse liver. Biochem J (1989) 263:355–63 2556992 10.1042/bj2630355 PMC1133437

[46] Chou HH Takematsu H Diaz S Iber J Nickerson E Wright KL A mutation in human CMP-sialic acid hydroxylase occurred after the Homo-Pan divergence. Proc Natl Acad Sci U S A (1998) 95:11751–6 10.1073/pnas.95.20.11751 9751737 PMC21712

[47] Irie A Koyama S Kozutsumi Y Kawasaki T Suzuki A The molecular basis for the absence of N-glycolylneuraminic acid in humans. J Biol Chem (1998) 273:15866–71 10.1074/jbc.273.25.15866 9624188

[48] Hayakawa T Satta Y Gagneux P Varki A Takahata N Alu-mediated inactivation of the human CMP-N-acetylneuraminic acid hydroxylase gene. Proc Natl Acad Sci U S A (2001) 98:11399–404 10.1073/pnas.191268198 11562455 PMC58741

[49] Varki A Gagneux P Human-specific evolution of sialic acid targets: explaining the malignant malaria mystery? Proc Natl Acad Sci U S A (2009) 106:14739–40 10.1073/pnas.0908196106 19717444 PMC2736437

[50] Ghaderi D Springer SA Ma F Cohen M Secrest P Taylor RE Sexual selection by female immunity against paternal antigens can fix loss of function alleles. Proc Natl Acad Sci U S A (2011) 108:17743–8 10.1073/pnas.1102302108 21987817 PMC3203784

[51] Varki A Colloquium paper: uniquely human evolution of sialic acid genetics and biology. Proc Natl Acad Sci U S A (2010) 107(Suppl 2):8939–46 10.1073/pnas.0914634107 20445087 PMC3024026

[52] Wang X Mitra N Cruz P Deng L NISC Comparative Sequencing Program Varki N Evolution of siglec-11 and siglec-16 genes in hominins. Mol Biol Evol (2012) 29(8):2073–86 10.1093/molbev/mss077 22383531 PMC3408085

[53] Schauer R Srinivasan GV Coddeville B Zanetta JP Guerardel Y Low incidence of N-glycolylneuraminic acid in birds and reptiles and its absence in the platypus. Carbohydr Res (2009) 344:1494–500 10.1016/j.carres.2009.05.020 19541293

[54] Fujii Y Higashi H Ikuta K Kato S Naiki M Specificities of human heterophilic Hanganutziu and Deicher (H-D) antibodies and avian antisera against H-D antigen-active glycosphingolipids. Mol Immunol (1982) 19:87–94 6176858 10.1016/0161-5890(82)90250-4

[55] Miyoshi I Fujii Y Naiki M Avian antisera to various gangliosides: detection by enzyme immunoassay. J Biochem (1982) 92:89–94 6749835 10.1093/oxfordjournals.jbchem.a133935

[56] Naiki M Fujii Y Ikuta K Higashi H Kato S Expression of Hanganutziu and Deicher type heterophile antigen on the cell surface of Marek’s disease lymphoma. Adv Exp Med Biol (1982) 152:445–56 6182765

[57] Higashi H Ikuta K Ueda S Kato S Hirabayashi Y Matsumoto M Characterization of N-glycolyneuraminic acid-containing glycosphingolipids from a Marek’s disease lymphoma-derived chicken cell line, MSB1, as tumor-associated heterophile Hanganutziu-Deicher antigens. J Biochem (1984) 95:785–94 6202681 10.1093/oxfordjournals.jbchem.a134670

[58] Tangvoranuntakul P Gagneux P Diaz S Bardor M Varki N Varki A Human uptake and incorporation of an immunogenic nonhuman dietary sialic acid. Proc Natl Acad Sci U S A (2003) 100:12045–50 10.1073/pnas.2131556100 14523234 PMC218710

[59] Hedlund M Padler-Karavani V Varki NM Varki A Evidence for a human-specific mechanism for diet and antibody-mediated inflammation in carcinoma progression. Proc Natl Acad Sci U S A (2008) 105:18936–41 10.1073/pnas.0803943105 19017806 PMC2596253

[60] Chen Y Pan L Liu N Troy FA Wang B LC-MS/MS quantification of N-acetylneuraminic acid, N-glycolylneuraminic acid and ketodeoxynonulosonic acid levels in the urine and potential relationship with dietary sialic acid intake and disease in 3- to 5-year-old children. Br J Nutr (2014) 111(2):332–41 10.1017/S0007114513002468 23915700

[61] Bardor M Nguyen DH Diaz S Varki A Mechanism of uptake and incorporation of the non-human sialic acid N-glycolylneuraminic acid into human cells. J Biol Chem (2005) 280:4228–37 10.1074/jbc.M412040200 15557321

[62] Nguyen DH Tangvoranuntakul P Varki A Effects of natural human antibodies against a nonhuman sialic acid that metabolically incorporates into activated and malignant immune cells. J Immunol (2005) 175:228–36 15972653 10.4049/jimmunol.175.1.228

[63] Nohle U Beau JM Schauer R Uptake, metabolism and excretion of orally and intravenously administered, double-labeled N-glycoloylneuraminic acid and single-labeled 2-deoxy-2,3-dehydro-N-acetylneuraminic acid in mouse and rat. Eur J Biochem (1982) 126:543–8 10.1111/j.1432-1033.1982.tb06815.x 7140746

[64] Nohle U Schauer R Uptake, metabolism and excretion of orally and intravenously administered, 14C- and 3H-labeled N-acetylneuraminic acid mixture in the mouse and rat. Hoppe Seylers Z Physiol Chem (1981) 362:1495–506 10.1515/bchm2.1981.362.2.1495 7030915

[65] Nohle U Schauer R Metabolism of sialic acids from exogenously administered sialyllactose and mucin in mouse and rat. Hoppe Seylers Z Physiol Chem (1984) 365:1457–67 10.1515/bchm2.1984.365.2.1457 6526381

[66] Hedlund M Tangvoranuntakul P Takematsu H Long JM Housley GD Kozutsumi Y N-glycolylneuraminic acid deficiency in mice: implications for human biology and evolution. Mol Cell Biol (2007) 27:4340–6 10.1128/MCB.00379-07 17420276 PMC1900035

[67] Banda K Gregg CJ Chow R Varki NM Varki A Metabolism of vertebrate amino sugars with N-glycolyl groups: mechanisms underlying gastrointestinal incorporation of the non-human sialic acid xeno-autoantigen N-glycolylneuraminic acid. J Biol Chem (2012) 287:28852–64 10.1074/jbc.M112.364182 22692204 PMC3436511

[68] Bergfeld AK Pearce OM Diaz SL Pham T Varki A Metabolism of vertebrate amino sugars with N-glycolyl groups: elucidating the intracellular fate of the non-human sialic acid N-glycolylneuraminic acid. J Biol Chem (2012) 287:28865–81 10.1074/jbc.M112.363549 22692205 PMC3436522

[69] Vamecq J Poupaert JH Studies on the metabolism of glycolyl-CoA. Biochem Cell Biol (1990) 68:846–51 10.1139/o90-125 1976013

[70] Vamecq J Mestdagh N Henichart J-P Poupaert J Subcellular distribution of glycolyltransferases in rodent liver and their significance in special reference to the synthesis of N-glycolylneuraminic acid. J Biochem (1992) 111:579–83 1639752 10.1093/oxfordjournals.jbchem.a123800

[71] Zhu A Hurst R Anti-N-glycolylneuraminic acid antibodies identified in healthy human serum. Xenotransplantation (2002) 9:376–81 10.1034/j.1399-3089.2002.02138.x 12371933

[72] Padler-Karavani V Yu H Cao H Chokhawala H Karp F Varki N Diversity in specificity, abundance, and composition of anti-Neu5Gc antibodies in normal humans: potential implications for disease. Glycobiology (2008) 18:818–30 10.1093/glycob/cwn072 18669916 PMC2586336

[73] Padler-Karavani V Hurtado-Ziola N Pu M Yu H Huang S Muthana S Human xeno-autoantibodies against a non-human sialic acid serve as novel serum biomarkers and immunotherapeutics in cancer. Cancer Res (2011) 71:3352–63 10.1158/0008-5472.CAN-10-4102 21505105 PMC3085609

[74] Asaoka H Nishinaka S Wakamiya N Matsuda H Murata M Two chicken monoclonal antibodies specific for heterophil Hanganutziu-Deicher antigens. Immunol Lett (1992) 32:91–6 1379980 10.1016/0165-2478(92)90205-3

[75] Malykh YN Schauer R Shaw L N-glycolylneuraminic acid in human tumours. Biochimie (2001) 83:623–34 10.1016/S0300-9084(01)01303-7 11522391

[76] Iznaga N Carr A Fernández LE Solozabal J Núñez G Perdomo Y Amplified ELISA to detect autoantibodies to N-glycolyl-GM3 ganglioside. J Clin Lab Immunol (1996) 48:75–85 16296265

[77] Padlan EA Kabat EA Model-building study of the combining sites of two antibodies to alpha(1–6)dextran. Proc Natl Acad Sci U S A (1988) 85:6885–9 10.1073/pnas.85.18.6885 2457920 PMC282083

[78] Chang WW Yu CY Lin TW Wang PH Tsai YC Soyasaponin I decreases the expression of alpha2,3-linked sialic acid on the cell surface and suppresses the metastatic potential of B16F10 melanoma cells. Biochem Biophys Res Commun (2006) 341:614–9 10.1016/j.bbrc.2005.12.216 16427612

[79] Yu H Chokhawala HA Varki A Chen X Efficient chemoenzymatic synthesis of biotinylated human serum albumin-sialoglycoside conjugates containing O-acetylated sialic acids. Org Biomol Chem (2007) 5:2458–63 10.1039/b706507h 17637967 PMC2769491

[80] Usuba O Fujii Y Miyoshi I Naiki M Sendo F Establishment of a human monoclonal antibody to Hanganutziu-Deicher antigen as a tumor-associated carbohydrate antigen. Jpn J Cancer Res (1988) 79:1340–8 10.1111/j.1349-7006.1988.tb01565.x 3148605 PMC5917656

[81] Sanai Y Yamasaki M Nagai Y Monoclonal antibody directed to a Hanganutziu-Deicher active ganglioside, GM2 (NeuGc). Biochim Biophys Acta (1988) 958:368–74 10.1016/0005-2760(88)90222-6 2449247

[82] Asaoka H Matsuda H Detection of N-glycolylneuraminic acid-containing glycoproteins from various animal erythrocytes by chicken monoclonal antibody against Hanganutziu-Deicher antigens. J Vet Med Sci (1994) 56:375–7 10.1292/jvms.56.375 8075229

[83] Koda T Shimosakoda T Nishinaka S Asaoka H Nakaba H Tamura I Diagnosis of hepatocellular carcinoma using chicken anti-monoclonal antibody – as tumor marker on the heterophilic Hanganutziu-Deicher antigen. Gan To Kagaku Ryoho (1994) 21:2771–7 7993113

[84] Watarai S Kushi Y Shigeto R Misawa N Eishi Y Handa S Production of monoclonal antibodies directed to Hanganutziu-Deicher active gangliosides, N-glycolylneuraminic acid-containing gangliosides. J Biochem (1995) 117:1062–9 8586620 10.1093/oxfordjournals.jbchem.a124807

[85] Pham T Gregg CJ Karp F Chow R Padler-Karavani V Cao H Evidence for a novel human-specific xeno-auto-antibody response against vascular endothelium. Blood (2009) 114:5225–35 10.1182/blood-2009-05-220400 19828701 PMC2792214

[86] Taylor RE Gregg CJ Padler-Karavani V Ghaderi D Yu H Huang S Novel mechanism for the generation of human xeno-autoantibodies against the nonhuman sialic acid N-glycolylneuraminic acid. J Exp Med (2010) 207:1637–46 10.1084/jem.20100575 20624889 PMC2916132

[87] Pan A Sun Q Bernstein AM Schulze MB Manson JE Willett WC Red meat consumption and risk of type 2 diabetes: 3 cohorts of US adults and an updated meta-analysis. Am J Clin Nutr (2011) 94:1088–96 10.3945/ajcn.111.018978 21831992 PMC3173026

[88] Micha R Wallace SK Mozaffarian D Red and processed meat consumption and risk of incident coronary heart disease, stroke, and diabetes mellitus: a systematic review and meta-analysis. Circulation (2010) 121:2271–83 10.1161/CIRCULATIONAHA.109.924977 20479151 PMC2885952

[89] Pan A Sun Q Bernstein AM Schulze MB Manson JE Stampfer MJ Red meat consumption and mortality: results from 2 prospective cohort studies. Arch Intern Med (2012) 172:555–63 10.1001/archinternmed.2011.2287 22412075 PMC3712342

[90] World Cancer Research Fund International Food, Nutrition, Physical Activity and the Prevention of Cancer: A Global Perspective. Washington, DC: American Institute for Cancer Research (2007).

[91] Gonzalez CA Riboli E Diet and cancer prevention: contributions from the European Prospective Investigation into Cancer and Nutrition (EPIC) Study. Eur J Cancer (2010) 46:2555–62 10.1016/j.ejca.2010.07.025 20843485

[92] Arem H Gunter MJ Cross AJ Hollenbeck AR Sinha R A prospective investigation of fish, meat and cooking-related carcinogens with endometrial cancer incidence. Br J Cancer (2013) 109:756–60 10.1038/bjc.2013.252 23695021 PMC3738127

[93] Salehi M Moradi-Lakeh M Salehi MH Nojomi M Kolahdooz F Meat, fish, and esophageal cancer risk: a systematic review and dose-response meta-analysis. Nutr Rev (2013) 71:257–67 10.1111/nure.12028 23590703

[94] Zhu H Yang X Zhang C Zhu C Tao G Zhao L Red and processed meat intake is associated with higher gastric cancer risk: a meta-analysis of epidemiological observational studies. PLoS One (2013) 8:e70955 10.1371/journal.pone.0070955 23967140 PMC3743884

[95] Hori S Butler E McLoughlin J Prostate cancer and diet: food for thought? BJU Int (2011) 107:1348–59 10.1111/j.1464-410X.2010.09897.x 21518228

[96] Giovannucci E Rimm EB Stampfer MJ Colditz GA Ascherio A Willett WC Intake of fat, meat, and fiber in relation to risk of colon cancer in men. Cancer Res (1994) 54:2390–7 8162586

[97] Stavric B Biological significance of trace levels of mutagenic heterocyclic aromatic amines in human diet: a critical review. Food Chem Toxicol (1994) 32:977–94 10.1016/0278-6915(94)90093-0 7959450

[98] Santarelli RL Pierre F Corpet DE Processed meat and colorectal cancer: a review of epidemiologic and experimental evidence. Nutr Cancer (2008) 60:131–44 10.1080/01635580701684872 18444144 PMC2661797

[99] Bastide NM Pierre FH Corpet DE Heme iron from meat and risk of colorectal cancer: a meta-analysis and a review of the mechanisms involved. Cancer Prev Res (Phila) (2011) 4:177–84 10.1158/1940-6207.CAPR-10-0113 21209396

[100] Koeth RA Wang Z Levison BS Buffa JA Org E Sheehy BT Intestinal microbiota metabolism of L-carnitine, a nutrient in red meat, promotes atherosclerosis. Nat Med (2013) 19:576–85 10.1038/nm.3145 23563705 PMC3650111

[101] Tang WH Wang Z Levison BS Koeth RA Britt EB Fu X Intestinal microbial metabolism of phosphatidylcholine and cardiovascular risk. N Engl J Med (2013) 368:1575–84 10.1056/NEJMoa1109400 23614584 PMC3701945

[102] Lopez-Garcia E Schulze MB Fung TT Meigs JB Rifai N Manson JE Major dietary patterns are related to plasma concentrations of markers of inflammation and endothelial dysfunction. Am J Clin Nutr (2004) 80:1029–35 15447916 10.1093/ajcn/80.4.1029

[103] Mantovani A Romero P Palucka AK Marincola FM Tumour immunity: effector response to tumour and role of the microenvironment. Lancet (2008) 371:771–83 10.1016/S0140-6736(08)60241-X 18275997

[104] Schreiber RD Old LJ Smyth MJ Cancer immunoediting: integrating immunity’s roles in cancer suppression and promotion. Science (2011) 331:1565–70 10.1126/science.1203486 21436444

[105] Boon T Cerottini J-C Van den Eynde B van der Bruggen P Van Pel A Tumor antigens recognized by T lymphocytes. Annu Rev Immunol (1994) 12:337–65 8011285 10.1146/annurev.iy.12.040194.002005

[106] Mellman I Coukos G Dranoff G Cancer immunotherapy comes of age. Nature (2011) 480:480–9 10.1038/nature10673 22193102 PMC3967235

[107] Pardoll D Does the immune system see tumors as foreign or self? Annu Rev Immunol (2003) 21:807–39 12615893 10.1146/annurev.immunol.21.120601.141135

[108] Finn OJ Cancer immunology. N Engl J Med (2008) 358:2704–15 10.1056/NEJMra072739 18565863

[109] Coussens LM Werb Z Inflammation and cancer. Nature (2002) 420:860–7 10.1038/nature01322 12490959 PMC2803035

[110] Hanahan D Coussens LM Accessories to the crime: functions of cells recruited to the tumor microenvironment. Cancer Cell (2012) 21:309–22 10.1016/j.ccr.2012.02.022 22439926

[111] Gabrilovich DI Nagaraj S Myeloid-derived suppressor cells as regulators of the immune system. Nat Rev Immunol (2009) 9:162–74 10.1038/nri2506 19197294 PMC2828349

[112] Wolchok JD Kluger H Callahan MK Postow MA Rizvi NA Lesokhin AM Nivolumab plus ipilimumab in advanced melanoma. N Engl J Med (2013) 369(2):122–33 10.1056/NEJMoa1302369 23724867 PMC5698004

[113] Restifo NP Dudley ME Rosenberg SA Adoptive immunotherapy for cancer: harnessing the T cell response. Nat Rev Immunol (2012) 12:269–81 10.1038/nri3191 22437939 PMC6292222

[114] Rosenberg SA Dudley ME Cancer regression in patients with metastatic melanoma after the transfer of autologous antitumor lymphocytes. Proc Natl Acad Sci U S A (2004) 101(Suppl 2):14639–45 10.1073/pnas.0405730101 15381769 PMC521998

[115] Coussens LM Zitvogel L Palucka AK Neutralizing tumor-promoting chronic inflammation: a magic bullet? Science (2013) 339:286–91 10.1126/science.1232227 23329041 PMC3591506

[116] Balkwill F Mantovani A Inflammation and cancer: back to Virchow? Lancet (2001) 357:539–45 10.1016/S0140-6736(00)04046-0 11229684

[117] Grivennikov SI Greten FR Karin M Immunity, inflammation, and cancer. Cell (2010) 140:883–99 10.1016/j.cell.2010.01.025 20303878 PMC2866629

[118] Wang D DuBois RN Inflammatory mediators and nuclear receptor signaling in colorectal cancer. Cell Cycle (2007) 6:682–5 10.4161/cc.6.6.4030 17374999

[119] Rothwell PM Fowkes FG Belch JF Ogawa H Warlow CP Meade TW Effect of daily aspirin on long-term risk of death due to cancer: analysis of individual patient data from randomised trials. Lancet (2011) 377:31–41 10.1016/S0140-6736(10)62110-1 21144578

[120] Gupta RA Dubois RN Colorectal cancer prevention and treatment by inhibition of cyclooxygenase-2. Nat Rev Cancer (2001) 1:11–21 10.1038/35094017 11900248

[121] Elwood PC Gallagher AM Duthie GG Mur LA Morgan G Aspirin, salicylates, and cancer. Lancet (2009) 373:1301–9 10.1016/S0140-6736(09)60243-9 19328542

[122] Mantovani A Allavena P Sica A Balkwill F Cancer-related inflammation. Nature (2008) 454:436–44 10.1038/nature07205 18650914

[123] Joyce JA Pollard JW Microenvironmental regulation of metastasis. Nat Rev Cancer (2009) 9:239–52 10.1038/nrc2618 19279573 PMC3251309

[124] Psaila B Lyden D The metastatic niche: adapting the foreign soil. Nat Rev Cancer (2009) 9:285–93 10.1038/nrc2621 19308068 PMC3682494

[125] De Palma M Lewis CE Macrophage regulation of tumor responses to anticancer therapies. Cancer Cell (2013) 23:277–86 10.1016/j.ccr.2013.02.013 23518347

[126] Varki A Kannagi R Toole BP Glycosylation changes in cancer. In: Varki A Cummings RD Esko JD Freeze HH Stanley P Bertozzi CR, editors. Essentials of Glycobiology. Cold Spring Harbor, NY: Cold Spring Harbor Laboratory Press (2009). p. 617–32 20301279

[127] Yin J Hashimoto A Izawa M Miyazaki K Chen GY Takematsu H Hypoxic culture induces expression of sialin, a sialic acid transporter, and cancer-associated gangliosides containing non-human sialic acid on human cancer cells. Cancer Res (2006) 66:2937–45 10.1158/0008-5472.CAN-05-2615 16540641

[128] Padler-Karavani V Song X Yu H Hurtado-Ziola N Huang S Muthana S Cross-comparison of protein recognition of sialic acid diversity on two novel sialoglycan microarrays. J Biol Chem (2012) 287:22593–608 10.1074/jbc.M112.359323 22549775 PMC3391140

[129] Meyerhardt JA Niedzwiecki D Hollis D Saltz LB Hu FB Mayer RJ Association of dietary patterns with cancer recurrence and survival in patients with stage III colon cancer. JAMA (2007) 298:754–64 10.1001/jama.298.7.754 17699009

[130] Rabinovich GA Croci DO Regulatory circuits mediated by lectin-glycan interactions in autoimmunity and cancer. Immunity (2012) 36:322–35 10.1016/j.immuni.2012.03.004 22444630

[131] Fuster MM Esko JD The sweet and sour of cancer: glycans as novel therapeutic targets. Nat Rev Cancer (2005) 5:526–42 10.1038/nrc1649 16069816

[132] Yonezawa S Tachikawa T Shin S Sato E Sialosyl-Tn antigen. Its distribution in normal human tissues and expression in adenocarcinomas. Am J Clin Pathol (1992) 98:167–74 1510031 10.1093/ajcp/98.2.167

[133] Ogata S Koganty R Reddish M Longenecker BM Chen A Perez C Different modes of sialyl-Tn expression during malignant transformation of human colonic mucosa. Glycoconj J (1998) 15:29–35 10.1023/A:1006935331756 9530954

[134] Kobayashi H Terao T Kawashima Y Serum sialyl Tn as an independent predictor of poor prognosis in patients with epithelial ovarian cancer. J Clin Oncol (1992) 10:95–101 1727929 10.1200/JCO.1992.10.1.95

[135] Imai J Ghazizadeh M Naito Z Asano G Immunohistochemical expression of T, Tn and sialyl-Tn antigens and clinical outcome in human breast carcinoma. Anticancer Res (2001) 21:1327–34 11396208

[136] Kim GE Bae HI Park HU Kuan SF Crawley SC Ho JJ Aberrant expression of MUC5AC and MUC6 gastric mucins and sialyl Tn antigen in intraepithelial neoplasms of the pancreas. Gastroenterology (2002) 123:1052–60 10.1053/gast.2002.36018 12360467

[137] Conze T Carvalho AS Landegren U Almeida R Reis CA David L MUC2 mucin is a major carrier of the cancer-associated sialyl-Tn antigen in intestinal metaplasia and gastric carcinomas. Glycobiology (2010) 20:199–206 10.1093/glycob/cwp161 19815850

[138] Cao Y Stosiek P Springer GF Karsten U Thomsen-Friedenreich-related carbohydrate antigens in normal adult human tissues: a systematic and comparative study. Histochem Cell Biol (1996) 106:197–207 10.1007/BF02484401 8877380

[139] Marquina G Waki H Fernandez LE Kon K Carr A Valiente O Gangliosides expressed in human breast cancer. Cancer Res (1996) 56:5165–71 8912852

[140] Carr A Mullet A Mazorra Z Vázquez AM Alfonso M Mesa C A mouse IgG1 monoclonal antibody specific for N-glycolyl GM3 ganglioside recognized breast and melanoma tumors. Hybridoma (2000) 19:241–7 10.1089/02724570050109639 10952412

[141] Carr A Rodríguez E Arango Mdel C Camacho R Osorio M Gabri M Immunotherapy of advanced breast cancer with a heterophilic ganglioside (NeuGcGM3) cancer vaccine. J Clin Oncol (2003) 21:1015–21 10.1200/JCO.2003.02.124 12637465

[142] Fernández-Marrero Y Hernández T Roque-Navarro L Talavera A Moreno E Griñán T Switching on cytotoxicity by a single mutation at the heavy chain variable region of an anti-ganglioside antibody. Mol Immunol (2011) 48:1059–67 10.1016/j.molimm.2011.01.008 21306777

[143] Fernández-Marrero Y Roque-Navarro L Hernández T Dorvignit D Molina-Pérez M González A A cytotoxic humanized anti-ganglioside antibody produced in a murine cell line defective of N-glycolylated-glycoconjugates. Immunobiology (2011) 216:1239–47 10.1016/j.imbio.2011.07.004 21802167

[144] Scursoni AM Galluzzo L Camarero S Lopez J Lubieniecki F Sampor C Detection of N-glycolyl GM3 ganglioside in neuroectodermal tumors by immunohistochemistry: an attractive vaccine target for aggressive pediatric cancer. Clin Dev Immunol (2011) 2011:245181 10.1155/2011/245181 21941577 PMC3177098

[145] Scursoni AM Galluzzo L Camarero S Pozzo N Gabri MR de Acosta CM Detection and characterization of N-glycolyated gangliosides in Wilms tumor by immunohistochemistry. Pediatr Dev Pathol (2010) 13:18–23 10.2350/08-10-0544.1 19435393

[146] Blanco R Rengifo E Cedeno M Rengifo CE Alonso DF Carr A Immunoreactivity of the 14F7 Mab raised against N-Glycolyl GM3 ganglioside in epithelial malignant tumors from digestive system. ISRN Gastroenterol (2011) 2011:645641 10.5402/2011/645641 21991524 PMC3168460

[147] Blanco R Rengifo E Rengifo CE Cedeno M Frometa M Carr A Immunohistochemical reactivity of the 14F7 monoclonal antibody raised against N-glycolyl GM3 ganglioside in some benign and malignant skin neoplasms. ISRN Dermatol (2011) 2011:848909 10.5402/2011/848909 22363862 PMC3262530

[148] Blanco R Rengifo CE Cedeno M Frometa M Rengifo E Carr A Immunoreactivity of the 14F7 Mab (raised against N-Glycolyl GM3 ganglioside) as a positive prognostic factor in non-small-cell lung cancer. Patholog Res Int (2012) 2012:235418 10.1155/2012/235418 22482082 PMC3317082

[149] von Mensdorff-Pouilly S Petrakou E Kenemans P van Uffelen K Verstraeten AA Snijdewint FG Reactivity of natural and induced human antibodies to MUC1 mucin with MUC1 peptides and N-acetylgalactosamine (GalNAc) peptides. Int J Cancer (2000) 86:702–12 10.1002/(SICI)1097-0215(20000601)86:53.3.CO;2-T 10797294

[150] Huang ZH Shi L Ma JW Sun ZY Cai H Chen YX A totally synthetic, self-assembling, adjuvant-free MUC1 glycopeptide vaccine for cancer therapy. J Am Chem Soc (2012) 134:8730–3 10.1021/ja211725s 22587010

[151] Sliwkowski MX Mellman I Antibody therapeutics in cancer. Science (2013) 341:1192–8 10.1126/science.1241145 24031011

[152] Ghaderi D Zhang M Hurtado-Ziola N Varki A Production platforms for biotherapeutic glycoproteins. Occurrence, impact, and challenges of non-human sialylation. Biotechnol Genet Eng Rev (2012) 28:147–75 10.5661/bger-28-147 22616486

[153] Ghaderi D Taylor RE Padler-Karavani V Diaz S Varki A Implications of the presence of N-glycolylneuraminic acid in recombinant therapeutic glycoproteins. Nat Biotechnol (2010) 28:863–7 10.1038/nbt.1651 20657583 PMC3077421

[154] Padler-Karavani V Tremoulet AH Yu H Chen X Burns JC Varki A A simple method for assessment of human anti-Neu5Gc antibodies applied to Kawasaki disease. PLoS One (2013) 8:e58443 10.1371/journal.pone.0058443 23520510 PMC3592828

[155] Weinberg RA Supplement 11.7 How Does Diet Affect Colon Cancer Incidence? The Biology of Cancer. New York: Garland Publishing (2014).

[156] Steinberg D Thematic review series: the pathogenesis of atherosclerosis. An interpretive history of the cholesterol controversy, part V: the discovery of the statins and the end of the controversy. J Lipid Res (2006) 47:1339–51 10.1194/jlr.R600009-JLR200 16585781

[157] Hirabayashi Y Suzuki T Suzuki Y Taki T Matsumoto M Higashi H A new method for purification of anti-glycosphingolipid antibody. Avian anti-hematoside (NeuGc) antibody. J Biochem (1983) 94:327–30 6619119 10.1093/oxfordjournals.jbchem.a134350

[158] Diaz SL Padler-Karavani V Ghaderi D Hurtado-Ziola N Yu H Chen X Sensitive and specific detection of the non-human sialic acid N-glycolylneuraminic acid in human tissues and biotherapeutic products. PLoS One (2009) 4:e4241 10.1371/journal.pone.0004241 19156207 PMC2626223

[159] Koda T Aosasa M Asaoka H Nakaba H Matsuda H Application of tyramide signal amplification for detection of N-glycolylneuraminic acid in human hepatocellular carcinoma. Int J Clin Oncol (2003) 8:317–21 10.1007/s10147-003-0346-4 14586758

[160] Miyake M Ito M Hitomi S Ikeda S Taki T Kurata M Generation of two murine monoclonal antibodies that can discriminate N-glycolyl and N-acetyl neuraminic acid residues of GM2 gangliosides. Cancer Res (1988) 48:6154–60 3167861

[161] Ozawa H Kawashima I Tai T Generation of murine monoclonal antibodies specific for N-glycolylneuraminic acid-containing gangliosides. Arch Biochem Biophys (1992) 294:427–33 10.1016/0003-9861(92)90707-4 1567198

[162] Kawashima I Ozawa H Kotani M Suzuki M Kawano T Gomibuchi M Characterization of ganglioside expression in human melanoma cells: immunological and biochemical analysis. J Biochem (1993) 114:186–93 8262898 10.1093/oxfordjournals.jbchem.a124153

[163] Nakamura K Suzuki H Hirabayashi Y Suzuki A IV3_(NeuGcalpha2-8NeuGc)-Gg4Cer is restricted to CD4+ T cells producing interleukin-2 and a small population of mature thymocytes in mice. J Biol Chem (1995) 270:3876–81 10.1074/jbc.270.8.3876 7533156

[164] Manzi AE Diaz S Varki A High-pressure liquid chromatography of sialic acids on a pellicular resin anion-exchange column with pulsed amperometric detection: a comparison with six other systems. Anal Biochem (1990) 188:20–32 10.1016/0003-2697(90)90523-C 2221361

[165] Rohrer JS Analyzing sialic acids using high-performance anion-exchange chromatography with pulsed amperometric detection. Anal Biochem (2000) 283:3–9 10.1006/abio.2000.4643 10929801

[166] Hurum DC Rohrer JS Five-minute glycoprotein sialic acid determination by high-performance anion exchange chromatography with pulsed amperometric detection. Anal Biochem (2011) 419:67–9 10.1016/j.ab.2011.08.002 21872565

[167] van der Ham M Prinsen BH Huijmans JG Abeling NG Dorland B Berger R Quantification of free and total sialic acid excretion by LC-MS/MS. J Chromatogr B Analyt Technol Biomed Life Sci (2007) 848(2):251–7 10.1016/j.jchromb.2006.10.066 17123874

[168] Allevi P Femia EA Costa ML Cazzola R Anastasia M Quantification of N-acetyl- and N-glycolylneuraminic acids by a stable isotope dilution assay using high-performance liquid chromatography-tandem mass spectrometry. J Chromatogr A (2008) 1212:98–105 10.1016/j.chroma.2008.10.039 18952219

[169] Hara S Yamaguchi M Takemori Y Furuhata K Ogura H Nakamura M Determination of mono-O-acetylated N-acetylneuraminic acids in human and rat sera by fluorometric high-performance liquid chromatography. Anal Biochem (1989) 179:162–6 10.1016/0003-2697(89)90218-2 2757191

[170] Lacomba R Salcedo J Alegria A Jesus Lagarda M Barbera R Matencio E Determination of sialic acid and gangliosides in biological samples and dairy products: a review. J Pharm Biomed Anal (2010) 51:346–57 10.1016/j.jpba.2009.04.023 19481897

[171] Gong S Ren HL Tian RY Lin C Hu P Li YS A novel analytical probe binding to a potential carcinogenic factor of N-glycolylneuraminic acid by SELEX. Biosens Bioelectron (2013) 49:547–54 10.1016/j.bios.2013.05.024 23777704

