# Corrigendum: Involvement of a Non-Human Sialic Acid in Human Cancer

**DOI:** 10.3389/fonc.2014.00083

**Published:** 2014-04-22

**Authors:** Annie N. Samraj, Heinz Läubli, Nissi Varki, Ajit Varki

**Affiliations:** ^1^Departments of Medicine, Pathology and Cellular and Molecular Medicine, Glycobiology Research and Training Center, University of California San Diego, La Jolla, CA, USA

**Keywords:** Neu5Gc, sialic acid, antibodies, inflammation, tumor antigen, red meat

Antibodies 14F7 and the corresponding anti-idiotype 1E10 (racotumomab, misspelled as racotumumab) were mistakenly conflated in this review. 14F7 reacts with (Neu5Gc)GM3, was recently humanized (1), but has not yet been tested in clinical trials. Racotumomab induced a human anti-(Neu5Gc)GM3 immune response, which correlated with longer median survival in non-small cell lung cancer (2). The first clinical trial result using racotumomab was actually published in 2002 (3). A phase III trial testing racotumomab in advanced non-small cell lung cancer began in 2011, and is currently recruiting (NCT01460472). These studies do not consider dietary Neu5Gc intake and incorporation as a variable that could affect (Neu5Gc)GM3 expression by human cancers.

## Conflict of Interest Statement

The authors declare that the research was conducted in the absence of any commercial or financial relationships that could be construed as a potential conflict of interest.
